# Supercritical CO_2_ Extraction and Identification of Ginsenosides in Russian and North Korean Ginseng by HPLC with Tandem Mass Spectrometry

**DOI:** 10.3390/molecules25061407

**Published:** 2020-03-19

**Authors:** Mayya Razgonova, Alexander Zakharenko, Tai-Sun Shin, Gyuhwa Chung, Kirill Golokhvast

**Affiliations:** 1 SEC Nanotechnology, Engineering school, Far Eastern Federal University, 690091 Vladivostok, Russia; razgonova.mp@dvfu.ru (M.R.); droopy@mail.ru (K.G.); 2 N.I. Vavilov All-Russian Institute of Plant Genetic Resources, 190000 Saint Petersburg, Russia; 3 Division of Food and Nutrition, Chonnam National University, Gwangju 61186, Korea; shints@chonnam.ac.kr; 4 Department of Biotechnology, Chonnam National University, Yeosu 59626, Korea; chung@chonnam.ac.kr; 5 Pacific Institute of Geography, Far Eastern Branch of Russian Academy of Sciences, 690041 Vladivostok, Russia

**Keywords:** ginseng, supercritical extraction, HPLC-MS/MS, ginsenosides, bioactive substances

## Abstract

Ginseng roots, *Panax ginseng* C.A. Meyer, obtained from cultivated ginseng grown in the Kaesong province (North Korea) and Primorye (Russia) were extracted using the supercritical CO_2_ extraction method. The extracts were subsequently analyzed by high-performance liquid chromatography with tandem mass spectrometry identification. The results showed the spectral peaks of typical ginsenosides with some other minor groups, and major differences were observed between the spectra of the two ginseng samples. The use of a pressure of 400 bar and higher allowed an increase in the yield of ginsenosides in comparison with similar previous studies

## 1. Introduction

Supercritical fluid solvents represent interesting alternatives to conventional solvents for producing high-quality natural food products without toxic residues [1,2]. The introduction of supercritical fluid extraction (SFE) has led to a novel technology that is being continually developed [3,4]. High-pressure SFE can be used to produce natural thermolabile compounds, leaving no organic solvent residues in food products, which are commonly observed with conventional extraction methods using methanol and hexane. Easy solvent removal from the final product, high selectivity, and moderate temperatures during the extraction process are the major advantages of SFE, leading to a significant increase in research focused on its use in the food, cosmetic, and pharmacological industries.

SFE has been used for extracting many natural products, including the fruits of *Schisandra chinensis* [5], microalgae rich in polyunsaturated fatty acids [6,7], lutein from the microalgae *Scenedesmus almeriensis* [8], lipid extraction [9], nimbin from Neem tree seeds [10], antioxidants from coriander seeds [11], ginger oleoresin (turpentine) from ginger [12], essential oils from the leaves of *Juniperus rigida* [13], triterpenic acids from *Eucalyptus globulus* [14], and many other compounds from plant matrices.

Far Eastern ginseng *Panax ginseng* C.A. Meyer (*P. ginseng*) is a perennial plant that has been used for millennia in traditional oriental medicine. The most studied biologically active components of ginseng, ginsenosides, are a homologous series of triterpene saponins with different glycosylation profiles [15]. Ginsenosides have been reported to exhibit diverse positive effects, including antitumor, chemopreventive, immunomodulating, and antidiabetic effects [16,17,18].

However, because of the temperature instability of ginsenosides, the production and quality of *P. ginseng* extracts depend on the extraction method [19]. Conventional extraction methods require long extraction times and large solvent volumes, which can lead to the thermal destruction of biologically active compounds. In addition, additional filtration and/or concentration procedures are often required to remove solid residues [20]. Supercritical extraction is an ideal solution to preserve the extractable target in a non-toxic and efficient manner.

The generation of metabolic profiles is a difficult task during the analysis of biologically active substances contained in plant matrices. The identification of detected compounds is commonly achieved by high-performance liquid chromatography with tandem mass spectrometry (HPLC-MS/MS). Technological advances have allowed for an expansion of the range of analytes and, most importantly, made it possible to identify these new compounds by accurate mass analysis (sixth decimal weight accuracy) [21,22].

Liquid chromatography combined with tandem mass spectrometry using an electrospray ionization source (LC-ESI-MS/MS) is a powerful tool for analyzing ginsenosides. Ji et al. used this method to study the composition of *P. ginseng* roots, combining HPLC studies with MS analysis [23,24,25].

Kite et al. used HPLC-MS to investigate melon ginsenosides and verify their authenticity [26], and Morinaga et al. identified ginsenosides in the pulp of American ginseng berries.

For compound identification, the elemental composition of the metabolite must be determined from high-accuracy mass data within 5 ppm of the theoretical mass [27]. It should be noted that a single mass value may correspond to more than one ginsenoside. Previously, more than 136 different ginsenosides with 62 unique elemental compositions have been identified in a single study [28].

## 2. Results and Discussion

**Figure 1:**
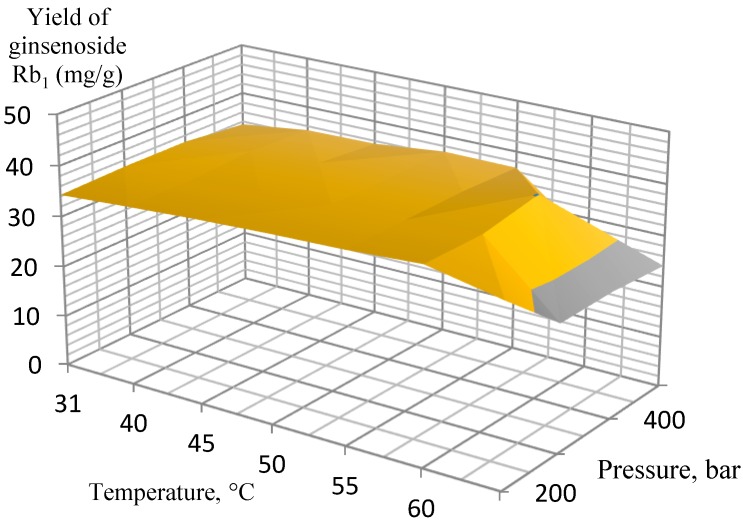
Orthogonal projection representing the results of extraction of ginsenoside Rb_1_ at a 200 to 500 bar and 3.4% EtOH co-solvent.

**Figure 2:**
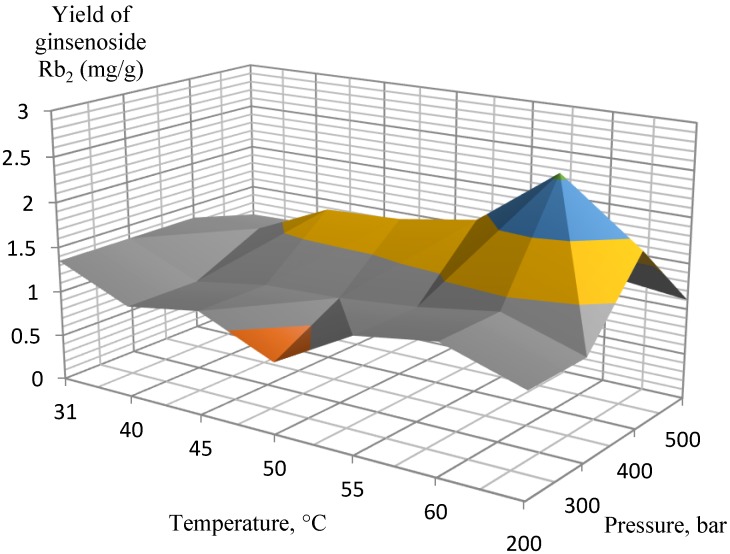
Orthogonal projection representing the results of extraction of ginsenoside Rb_2_ at 200 to 500 bar and 3.4% EtOH co-solvent.

**Figure 3:**
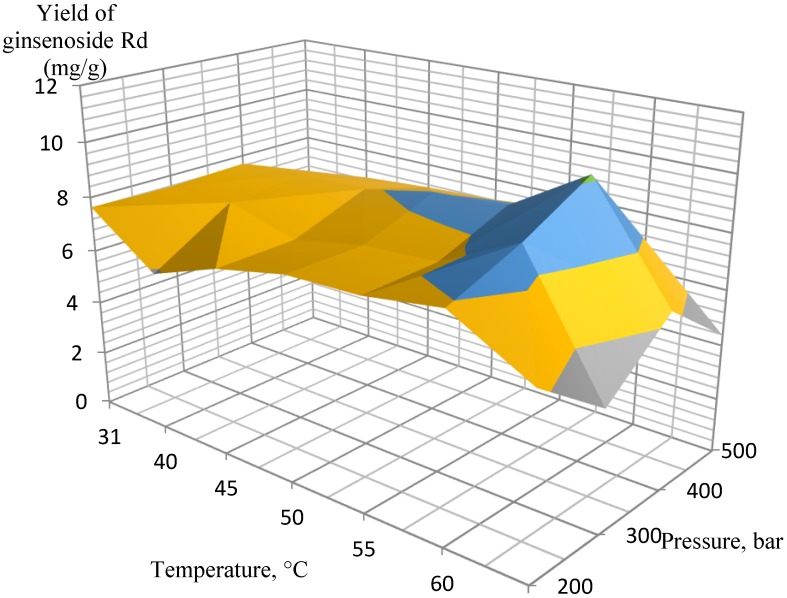
Orthogonal projection representing the results of extraction of ginsenoside Rd at 200 to 500 bar and 3.4% EtOH co-solvent.

**Figure 4:**
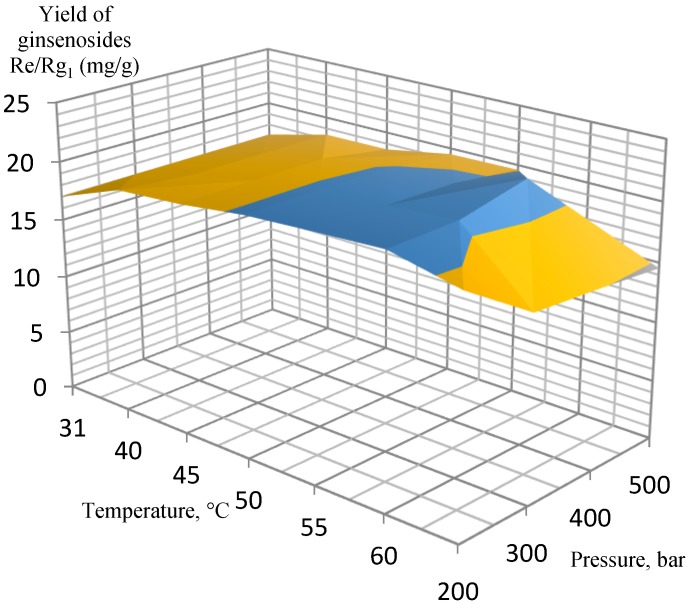
Orthogonal projection representing the results of extraction of ginsenosides Re and Rg_1_ at 200 to 500 bar and 3.4% EtOH co-solvent.

Several experimental conditions were investigated in the pressure range of 200–500 bar, 3.4% of co-solvent (ethanol, EtOH) in the liquid phase at 31–70 °C. After testing a wide range of pressures and temperatures, the most efficient extraction conditions were determined for extracting the target analytes from the ginseng roots. According to the experimental data, the orthogonal projections of the graphs were constructed separately for ginsenosides Rb_1_, Rb_2_, Rd, and Rg_1_/Re (Figure 1, Figure 2, Figure 3 and Figure 4, respectively).

These ginsenosides were chosen because their quantity and ratio were previously shown to be an effective marker for determining quality from different species, geographic environments, and cultivation cultures [29].

It is well known that the disadvantage of using pure CO_2_ for extraction and fractionation is that there is no pure dipole moment, and CO_2_ is an ineffective solvent for highly polar materials. To overcome this drawback, modifiers (ethanol, methanol, or n-hexane) can be used to increase the overall polarity of the liquid phase during extraction. In addition, modifiers allow for a more efficient extraction of solid materials by disrupting the interactions between the solutes and solid matrix. Many researchers have reported this synergistic effect in previous studies using supercritical CO_2_ extraction [30,31]. In spite of the fact that the co-solvent methanol is most often used for qualitative analysis, we chose ethanol as the co-solvent since extraction in a closed-cycle plant was carried out in this work, and the main idea was not just a qualitative analysis, but an analysis of the applicability of technology for the food industry [19,32,33]. Moreover, we decided to use the minimum amount of co-solvent, which gave a significant increase in the yield of the product. On the one hand, the further addition of the amount of a co-solvent shifted the system too much from the supercritical state since for ethanol the supercritical state occurs above 240 °C, and on the other hand, the further addition of ethanol did not give a significant increase in the extraction yield.

The extraction results for the isolated ginsenosides separately supported the initial conclusion that the optimal extraction conditions were 400 bar at 60 °C. When these parameters were achieved, a substantial increase in extract yields occurred, while a further increase in pressure and temperature did not affect the yields so significantly, and, therefore, it was not economical. In particular, this conclusion is the most pronounced in Figure 2 (ginsenoside Rb_2_ extraction) and Figure 3 (ginsenoside Rd extraction).

Obtaining chemical profiles is extremely important for the analysis of biological systems. The most commonly used methods in this regard are nuclear magnetic resonance (NMR) and HPLC-MS. Herein, HPLC-ESI-MS/MS with additional ionization and analysis of fragmented ions was used to obtain chemical profiles. High-accuracy mass spectrometric data were recorded using an ion trap amaZon SL equipped with an ESI source in negative ion mode with two-stage ion separation (MS/MS mode).

**Figure 5:**
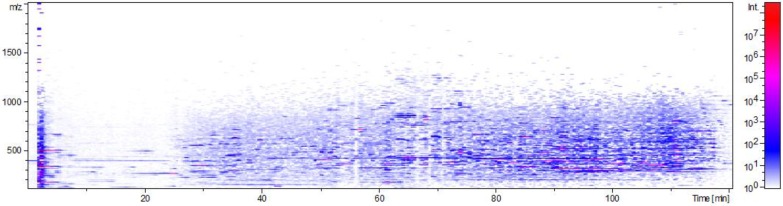
The distribution density of the analyzed chemical profiles in the ion chromatogram of the wild ginseng supercritical CO_2_ extract (Russia).

Figure 5 shows the distribution density of the analyzed chemical profiles in the ion chromatogram of the wild ginseng supercritical CO_2_ extract from Russia (HPLC ESI MS/MS). Visually, a rather high-density distribution of the target analytes in the analyzed extract was observed.

**Figure 6:**
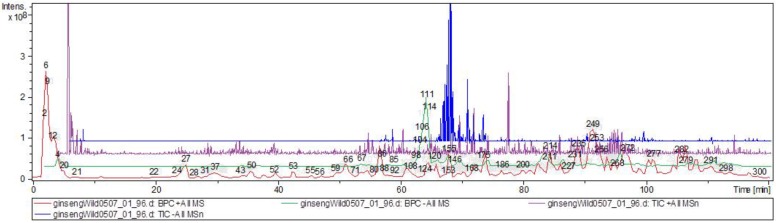
Representative chemical profiles of the wild ginseng’s (Russia) total ion chromatogram from the supercritical CO_2_ extract.

The chemical profiles of all the samples were obtained by HPLC-ESI-MS/MS. A total of 300 peaks were detected in the chromatogram (Figure 6), and 28 components were authenticated as ginsenosides by comparing the retention times, m/z values, and fragment ions with the literature data [34,35,36,37,38,39,40,41,42].

**Figure 7:**
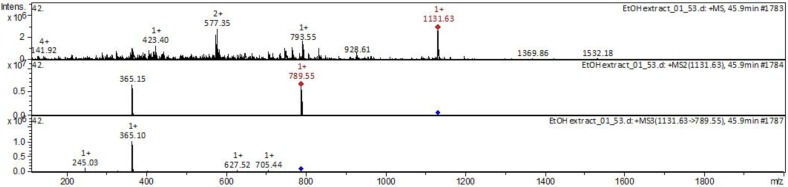
CID (collision-induced dissociation) spectra of ginsenoside Rb1 from wild ginseng (Russia), *m*/*z* 1131.63.

The collision-induced dissociation (CID) spectrum obtained in positive ion mode for triterpene glycoside ginsenoside Rb_1_ from Russian *P. ginseng* is shown in Figure 7.

**Figure 8:**
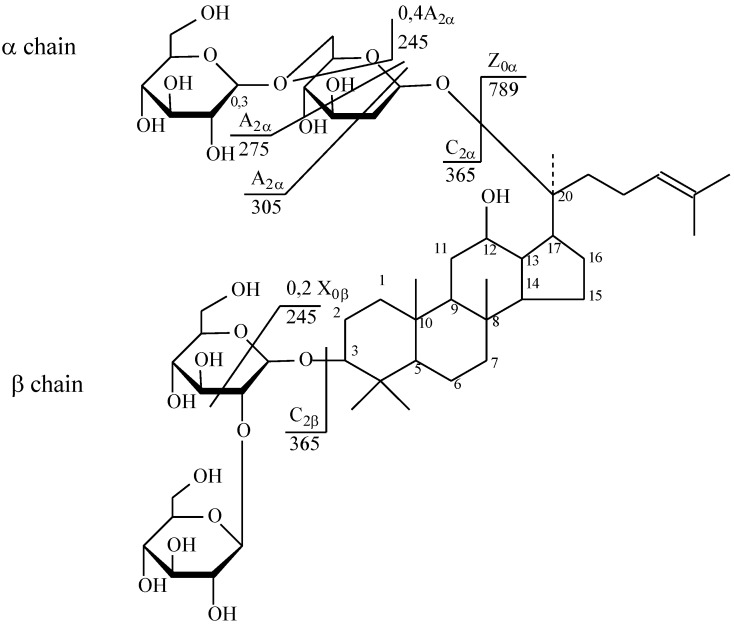
Structure of ginsenoside Rb_1_ from wild ginseng (Russia).

The [M + Na]^+^ ion produced two fragments—Z_0α at m/z 789.55 via loss of two hexose residues and dihexose C_2α at m/z 365.10 because of the higher reactivity of C20 composed to C3 (Figure 8). Z_0α also yielded a daughter ion at m/z 365.10 (C_2β), and the mass difference of 424.45 Da between the m/z 789.55 and 365.10 ions corresponded to the mass of panaxadiol with the loss of two water molecules. The C_2β ion mainly produced X_0β (m/z 245.03) by cross-ring cleavage, indicating that the β-chain consisted of two hexoses connected to each other at the 1,2 position. Thus, it was clarified that the hydrolysis of the oligosaccharide residue first occurs at the C-20 aglycone [40].

**Figure 9:**
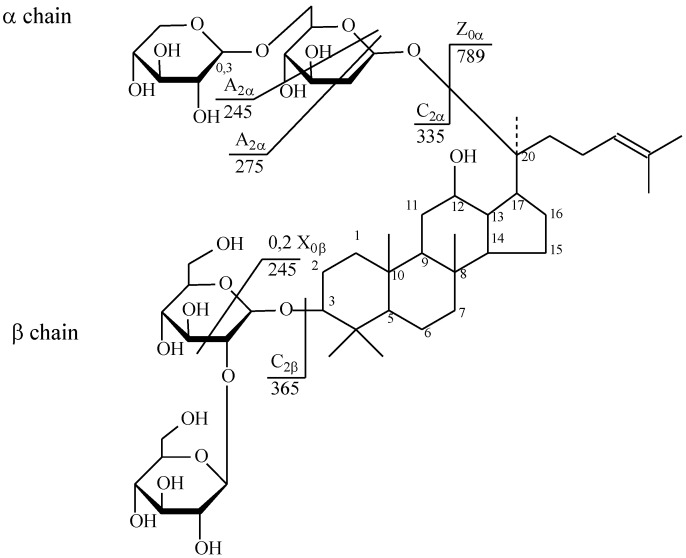
Structure of ginsenoside Rb_2_ from wild ginseng (Russia).

The fragmentation of ginsenoside Rb_2_ proceeded similarly, and its structure is shown in Figure 9.

**Figure 10:**
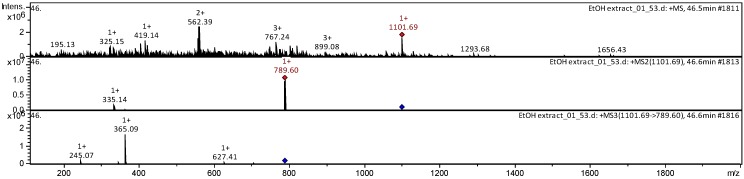
CID spectrum of the ginsenoside Rb2 from wild ginseng (Russia), *m*/*z* 1101.69.

The positive ion mode CID spectra of triterpene glycoside ginsenoside Rb_2_ from Russian *P. ginseng* is shown in Figure 10.

The molecular masses of the target analytes presented in the supercritical extract of wild ginseng *P. ginseng* (Russia) and analyzed by HPLC with tandem mass spectrometry are listed in Table 1 for easy identification.

A total of 27 ginsenosides were isolated from wild ginseng (Russia) via column chromatography and mass spectrometry. The structures were elucidated via stepwise ion fragmentation MS/MS and compared with the typical structural features from the literature data. In the wild ginseng, pairs of 20 R/S isomers, i.e., 20 R/S–acetyl Rg_1_, 20 R/S–Rh_1_, 20 R/S–Rf, 20 R/S–Rg_3_, and 20 R/S–Rh_2_, were isolated. This series of 20R ginsenosides, which are not often found in wild ginseng, are most likely obtained by the attack on the hydroxyl group at C-20 after selective deglycosylation (Kang et al., 2007). Ginsenosides Rg_9_, Rk_2_, and Rk_3_ and hydroxylases at C-25, i.e., 25–OH–20 R/S–Rh_4_, were also obtained. These compounds could be prepared by combining dehydration and hydration during heating and acid or enzymatic hydrolysis [43]. Thus, exhaustive supercritical extraction using ethanol could be used to obtain ginsenoside extracts from the ginseng crown. As well as using methanol as a co-solvent, it allows one to achieve good qualitative and quantitative indicators, while the use of ethanol in the food industry is more preferable compared to methanol [19,32]. We isolated 21 ginsenosides, which is a quarter more than previously isolated under supercritical extraction conditions; we attribute this to two factors. The first is that the conditions for the growth of ginseng in the northern regions are more contrasting in temperature, and the second, that we used pressures of up to 500 bar, while previously the researchers were limited to 220 bars [32,33].

The molecular masses of the target analytes isolated from the supercritical extract of ginseng *P. ginseng* (North Korea, Kaesong) are listed in Table 2 for easy identification.

A total of 15 ginsenosides were isolated from Korean ginseng (North Korea, Kaesong) via column chromatography and MS. In the Korean ginseng, pairs of 20 R/S isomers, i.e., 20 R/S–Rh_1_, 20 R/S–Rh_2_, 20 R/S–Rf, 20 R/S–Rg_2_, 20 R/S–Rg_3_, and 20 R/S–Rf_2_, were isolated. Ginsenosides that were hydrolyzed at C-25, i.e., 25–OH–20 R/S–Rh_1_, were also found. These compounds could be prepared by combining dehydration and hydration during heating and acid or enzymatic hydrolysis [43]. Oleanolic acid pentaterpene glycosides chikusetsusaponin IV A and silphioside G were also isolated (Table 2).

## 3. Materials and Methods

### 3.1. Materials

Samples of *P. ginseng* were purchased from the Lazovsky district of Primorye, Russia, and cultivated ginseng (*P. ginseng*) was obtained from the province of Kaesong, North Korea. All samples were morphologically authenticated according to the current Russian Pharmacopeia standards [44].

### 3.2. Chemicals and Reagents

HPLC-grade acetonitrile was purchased from Fisher Scientific (Southborough, UK), and MS-grade formic acid was obtained from Sigma-Aldrich (Steinheim, Germany). Ultra-pure water was prepared using a SIEMENS ULTRA clear instrument (SIEMENS Water Technologies, Berlin, Germany), and all other chemicals were of analytical grade.

### 3.3. Liquid Chromatography

HPLC was performed using a Shimadzu LC-20 Prominence system (Shimadzu, Kyoto Japan) equipped with ZORBAX Eclipse XDB C18 (150 × 4.6 mm, particle size: 5 μm) reverse-phase column for the separation of the multicomponent mixtures. The gradient elution program was as follows: 0.01–4 min, 100% A; 4–60 min, 100–25% A; 60–75 min, 25–0% A; control washing 75–120 min, 0% A. Solvent A - deionized water, solvent B - acetonitrile. The entire HPLC analysis was performed using a UV-VIS detector SPD-20A (Shimadzu, Kyoto Japan) at wavelengths of 230 and 330 nm at 17 °C provided with column oven CTO-20A (Shimadzu, Kyoto Japan) with an injection volume of 20 μL.

### 3.4. Supercritical Fluid Extraction

Supercritical CO_2_ extraction was performed using the TharSCF SFE-500 system (Waters, Pittsburgh, PA, USA) supercritical pressure extraction apparatus. System options include a co-solvent pump (Thar Waters P-50 High-Pressure Pump) for extracting polar samples; CO_2_ flow meter (Siemens, Berlin, Germany) to measure the amount of CO_2_ being supplied to the system; multiple extraction vessels to extract different sample sizes or to increase the throughput of the system. The flow rate was 50 mL/min for liquid CO_2_ and 1.76 mL/min for EtOH. For extraction, samples of 10 g of ginseng root were used. The extraction time was counted after reaching the set pressure and equilibrium flow, and it was 6 h for each sample.

### 3.5. Mass Spectrometry

MS analysis was performed using an ion trap amaZon SL (BRUKER DALTONIKS, Berlin, Germany) equipped with an ESI source in negative ion mode. The optimized parameters were as follows: ionization source temperature, 70 °C; gas flow, 4 L/min; nebulizer gas (atomizer), 7.3 psi; capillary voltage, 4500 V; endplate bend voltage, 1500 V; fragmentation voltage, 280 V; collision energy, 60 eV. An ion trap was used in the m/z 100–1700 scan range for MS and MS/MS analyses. The capture rate was one spectrum/s for MS and two spectra/s for MS/MS. Data collection was controlled by Windows software for BRUKER DALTONIKS. All experiments were performed in triplicate, and a two-stage ion separation mode (MS/MS mode) was implemented.

## 4. Conclusions

Supercritical CO_2_ extraction is a soft extractive method that can be executed at fairly low temperatures with a very sparing effect on the plant matrix. In addition, it features easy removal of the solvent from the resulting extract and is environmentally friendly, yielding a richer extractive material.

Over the past decade, more than 400 novel ginsenoside compounds have been reported. The use of newly developed methods for studying the chemical structure of ginsenosides, including HPLC-MS/MS, can significantly improve the accuracy of identification methods, facilitating further discoveries of biologically active compounds from plant matrices.

Ginseng roots, *Panax ginseng* C.A. Meyer, obtained from cultivated ginseng grown in the Kaesong province (North Korea) and Primorye (Russia) were extracted using the supercritical CO_2_ extraction method. We chose the conditions for extraction that are most suitable for subsequent scaling for food industrial extraction. The optimal co-solvent ethanol at a concentration of 3.4% was selected. The optimum temperature was determined to be 60 °C. The results showed the spectral peaks of typical ginsenosides with some other minor groups, and major differences were observed between the spectra of the two ginseng samples.

## Figures and Tables

**Table 1:** Components identified from the supercritical extract of *P. ginseng* (Russia).

№	Identity	Molecular Formula	Adducts	MS (*m*/*z*)	MS2 (*m*/*z*)	MS3 (*m*/*z*)
	**Triterpene Glycosides (Dammarane Type)**					
1	Ginsenoside Rk_3_	C_36_H_60_O_8_	[M − H]^−^	619.21	421.22	229.06; 347.07; 403.19
2	Malonyl ginsenoside Rb_1_	C_57_H_94_O_26_	[M − H]^−^	1149.81	1107.65	459.31; 621.44; 783.46; 945.52
3	Malonyl ginsenoside Rb_1_ isomer	C_57_H_94_O_26_	[M − H]^−^	1193.7	1151.72	604.33; 826.59; 946.58; 1109.59
4	Ginsenoside Rg_1_	C_42_H_72_O_14_	[M − H + HCOOH]^−^	845.79	799.65	475.45; 637.61
5	Ginsenoside Rd isomer	C_48_H_82_O_18_	[M − H + HCOOH]^−^	991.83	945.73	391.43; 475.5; 637.62; 783.68
6	Ginsenoside Rg_6_	C_42_H_70_O_12_	[M + Na]^+^	765.41	405.39	171.07; 281.12
7	Acetyl ginsenoside Rg_1_ isomer	C_44_H_74_O_15_	[M + Na]^+^	841.55	661.5	481.53; 573.28; 643.32
8	Ginsenoside Rf	C_42_H_72_O_14_	[M − H]^−^	846.81	799.65	391.34; 475.46; 545.54; 637.55
9	(Yesanchinoside d isomer	C_44_H_74_O_15_	[M + Na]^+^	841.62	661.47	481.48; 541.46; 571.59; 601.27; 643.42
10	Ginsenoside Rb_1_	C_54_H_92_O_23_	[M − H]^−^	1107.88	783.7	621.57; 460.52
	Ginsenoside Rb_1_	C_54_H_92_O_23_	[M + Na]^+^	1131.63	789.55	245.03; 365.10; 627.52; 705.44
11	Ginsenoside Rd	C_48_H_82_O_18_	[M − H]^−^	945.93	783.65	621.63; 459.39
12	Ginsenoside 20-glc-Rf	C_48_H_82_O_19_	[M − H]^−^	961.84	915.76	292.31; 375.99; 459.51; 621.51; 783.75
13	Ginsenoside 25-OH-Rh_4_	C_36_H_62_O_9_	[M − H]^−^	637.6	239.14	
14	Ginsenoside 20(*R*)-Rh_1_	C_36_H_62_O_9_	[M − H]^−^	683.65	475.4	375.38
15	Ginsenoside 20(*S*)-Rh_1_	C_36_H_62_O_9_	[M − H]^−^	683.64	637.59	375.42; 475.48; 549.31
16	Ginsenoside Rc	C_53_H_90_O_22_	[M − H]^−^	1077.86	783.59	621.66
17	Ginsenoside Rb_2_	C_53_H_90_O_22_	[M + Na]^+^	1101.69	789.60	245.07; 365.09
18	Ginsenoside 20(*S*)-Rf	C_42_H_72_O_14_	[M + Na]^+^	800.94	782.93	474.96; 307.83
19	Ginsenoside Rk_2_	C_42_H_72_O_15_	[M + Na]^+^	663.20	543.26	287.04; 367.26; 499.21
20	3β,12β-dihydroxydammar-20(22)*E*,24-diene-6-o-β-d-xylopyranosyl-(1→2)-O-β-d-glucopyranoside (DHDXG)	C_42_H_70_O_12_	[M + Na]^+^	751.19	631.31	243.08; 367.12; 455.2; 587.21
21	Ginsenoside Rg9	C_42_H_72_O_13_	[M + Na]^+^	781.79	707.44	377.14; 671.18
	**Oleanolic Acid Pentaterpene Glycosides**					
22	Ro	C_48_H_76_O_19_	[M − H]^−^	955.57	793.41	455.29; 613.38; 731.42
23	Methyl ester Ro	C_49_H_78_O_19_	[M + Na]^+^	969.48	364.96	304.95
24	Chikusetsusaponin IVA	C_42_H_66_O_14_	[M − H]^−^	793.36	334.97	274.94
25	Methyl ester chikusetsusaponin IVA	C_42_H_66_O_14_	[M + Na]^+^	807.38	627.34	203.05; 285.14; 361.77; 488.93
26	Silphioside G	C_42_H_66_O_14_	[M − H]^−^	793.7	613.49	483.3
27	Zingibroside R_1_	C_42_H_66_O_14_	[M − H]^−^	793.56	481.43	275.07

**Table 2:** Components identified from the supercritical extract of *P. ginseng* (North Korea, Kaesong).

№	Identity	Molecular Formula	Adducts	MS (*m*/*z*)	MS2 (*m*/*z*)	MS3 (*m*/*z*)
**Triterpene Glycosides (Dammarane Type)**						
1	Ginsenoside 20(R)-Rh_1_	C_36_H_62_O_9_	[M − H]^−^	637.38	597.32	375.42; 475.48
2	Ginsenoside 20(S)-Rh_1_	C_36_H_62_O_9_	[M − H]^−^	637.39	597.33	375.42; 475.49
3	Ginsenoside 20(R)-Rh_2_	C_36_H_62_O_8_	[M − H]^−^	621.32	580.2	390.33
4	Ginsenoside 20(S)-Rh_2_	C_36_H_63_O_10_	[M − H]^−^	621.32	580.24	390.34
5	Ginsenoside 25-OH-(S)-Rh_1_	C_36_H_63_O_10_	[M − H]^−^	654.41	375.15	332.26
6	Ginsenoside Rg_1_	C_42_H_72_O_14_	[M + HCOO]^−^	845.26	501.18	485.17
7	Ginsenoside F_2_	C_42_H_72_O_13_	[M + H]^+^	785.55	783.6	375.27; 459.33; 537.37; 621.36
8	Ginsenoside 20(S)-Rf_2_	C_42_H_74_O_14_	[M − H]^−^	801.80	767.68	378.21; 671.55
9	Ginsenoside 20-glu-Rf	C_48_H_82_O_19_	[M − H]^−^	961.59	681.45	637.44; 357.14; 401.12 595.46
10	Ginsenoside 20(R/S)-Rg_2_	C_42_H_72_O_13_	[M − H]^−^	783.54	529.38	429.21
11	Ginsenoside 20(R/S)-Rg_3_	C_42_H_72_O_13_	[M − H]^−^	783.68	737.87	694.71
12	Ginsenoside 20(R/S)-Rf	C_42_H_72_O_14_	[M − H]^−^	799.80	544.49	227.21; 280.18; 379.14
13	Notoginsenoside Rw_2_	C_41_H_70_O_14_	[M + Na]^−^	809.81	544.50	227.21; 280.18; 379.15
**Oleanolic Acid Pentaterpene Glycosides**						
14	Silphioside G	C_42_H_66_O_14_	[M − H]^−^	793.49	613.3	407.21; 509.33
15	Chikusetsusaponin IVA	C_42_H_66_O_14_	[M + H]^+^	795.67	631.39	511.18

## References

[1] Subra P., Castellani S., Jestin P., Aoufi A. (1998). Extraction of β-carotene with supercritical fluids. J. Supercrit. Fluids.

[2] Casas L., Mantell C., Rodríguez M., Torres A., Macías F.A., De La Ossa E.M., Cardoso L.C. (2009). Extraction of natural compounds with biological activity from sunflower leaves using supercritical carbon dioxide. Chem. Eng. J..

[3] Herrero M., Mendiola J.A., Cifuentes A., Ibáñez E. (2010). Supercritical fluid extraction: Recent advances and applications. J. Chromatogr. A.

[4] Plaza M., Herrero M., Cifuentes A., Ibáñez E. (2009). Innovative natural functional ingredients from microalgae. J. Agric. Food Chem..

[5] Choi Y.H., Kim J., Jeon S.H., Yoo K.-P., Lee H.-K. (1998). Optimum SFE condition for lignans of *Schisandra chinensis* fruits. Chromatographia.

[6] Macías-Sánchez M.D., Serrano C.M., Rodríguez-Rodríguez M., De La Ossa E.M., Lubián L.M., Montero O. (2008). Extraction of carotenoids and chlorophyll from microalgae with supercritical carbon dioxide and ethanol as cosolvent. J. Sep. Sci..

[7] Gouveia L., Nobre B., Marcelo F., Mrejen S., Cardoso M., Palavra A., Mendes R. (2007). Functional food oil coloured by pigments extracted from microalgae with supercritical CO_2_. Food Chem..

[8] Mehariya S., Iovine A., Di Sanzo G., LaRocca V., Martino M., Leone G.P., Casella P., Karatza D., Marino T., Musmarra D. (2019). Supercritical fluid extraction of lutein from *Scenedesmus almeriensis*. Molecules.

[9] Sahena F., Zaidul I.S.M., Jinap S., Karim A.A., Abbas K.A., Norulaini N.A.N., Omar A.K.M. (2009). Application of supercritical CO_2_ in lipid extraction—A review. J. Food Eng..

[10] Tonthubthimthong P., Douglas P.L., Douglas S., Luewisutthichat W., Teppaitoon W., Pengsopa L.-E. (2004). Extraction of nimbin from neem seeds using supercritical CO_2_ and a supercritical CO_2_–methanol mixture. J. Supercrit. Fluids.

[11] Yepez B., Espinosa M., López S., Bolaños G. (2002). Producing antioxidant fractions from herbaceous matrices by supercritical fluid extraction. Fluid Phase Equilibria.

[12] Zancan K.C., Marques M.O.M., Petenate A.J., Meireles M.A. (2002). Extraction of ginger (*Zingiber officinale* Roscoe) oleoresin with CO_2_ and co-solvents: A study of the antioxidant action of the extracts. J. Supercrit. Fluids.

[13] Meng X., Li D.-W., Zhou D., Wang N., Liu Q., Fan S. (2016). Chemical composition, antibacterial activity and related mechanism of the essential oil from the leaves of *Juniperus rigida* Sieb. et Zucc against *Klebsiella pneumoniae*. J. Ethnopharmacol..

[14] De Melo M., Oliveira E., Silvestre A., Silva C.M. (2012). Supercritical fluid extraction of triterpenic acids from *Eucalyptus globulus* bark. J. Supercrit. Fluids.

[15] Elyakov G.B., Strigina L.I., Khorlin A.Y., Kochetkov H.K. (1962). Glycosides of ginseng (*Panax ginseng* C. A. Mey). Russ. Chem. Bull..

[16] Brekhman I., Dardymov I.V., Dobriakov I. (1966). On the pharmacology of individual glycosides from the roots of *Panax ginseng* C.A. Mey. Farmakol. Toksikol..

[17] Park E.-K., Shin Y.-W., Lee H.-U., Kim S.-S., Lee Y.-C., Lee B.-Y., Kim N.-H. (2005). Inhibitory effect of ginsenoside Rb1 and compound K on NO and prostaglandin E2 biosyntheses of RAW264.7 cells induced by lipopolysaccharide. Boil. Pharm. Bull..

[18] Bao C., Wang Y., Min H., Zhang M., Du X., Han R., Liu X. (2015). Combination of ginsenoside Rg1 and bone marrow mesenchymal stem cell transplantation in the treatment of cerebral ischemia reperfusion injury in rats. Cell. Physiol. Biochem..

[19] Wood J., Bernards M.A., Wan W.-K., Charpentier P.A. (2006). Extraction of ginsenosides from North American ginseng using modified supercritical carbon dioxide. J. Supercrit. Fluids.

[20] Wang L., Weller C.L. (2006). Recent advances in extraction of nutraceuticals from plants. Trends Food Sci. Technol..

[21] Kochkin D.V., Kachala V.V., Shashkov A.S., Chizhov A.O., Chirva V.Y., Nosov A.M. (2013). Malonyl-ginsenoside content of a cell-suspension culture of *Panax japonicus* var. repens. Phytochemistry.

[22] Stavrianidi A., Baygildiev T.M., Stekolshchikova E.A., Shpigun O.A., Rodin I.A. (2019). New approaches to the determination and group identification of physiologically active compounds in plant materials and commercial products by high-performance liquid chromatography–mass spectrometry. J. Anal. Chem..

[23] Ji Q.C., Harkey M.R., Henderson G.L., Gershwin M.E., Stern J.S., Hackman R.M. (2001). Quantitative determination of ginsenosides by high-performance liquid chromatography-tandem mass spectrometry. Phytochem. Anal..

[24] Ligor T., Ludwiczuk A., Wolski T., Buszewski B. (2005). Isolation and determination of ginsenosides in American ginseng leaves and root extracts by LC-MS. Anal. Bioanal. Chem..

[25] Wang X., Sakuma T., Asafu-Adjaye E., Shiu G.K. (1999). Determination of ginsenosides in plant extracts from *Panax ginseng* and *Panax quinquefolius* L. by LC/MS/MS. Anal. Chem..

[26] Kite G.C., Howes M.-J.R., Leon C.J., Simmonds M.S. (2003). Liquid chromatography/mass spectrometry of malonyl-ginsenosides in the authentication of ginseng. Rapid Commun. Mass Spectrom..

[27] Brown P.N. (2011). Determination of ginsenoside content in Asian and North American ginseng raw materials and finished products by high-performance liquid chromatography: Single-laboratory validation. J. AOAC Int..

[28] Buckingham J. (1993). Dictionary of Natural Products.

[29] Yang L., Li C.-L., Cheng Y.-Y., Tsai T.-H., Tsai T.-H. (2019). Development of a validated UPLC-MS/MS method for analyzing major ginseng saponins from various ginseng species. Molecules.

[30] Yeo S.-D., Park S.-J., Kim J.-W., Kim J.-C. (2000). Critical properties of carbon dioxide + methanol, + ethanol, + 1-propanol, and + 1-butanol. J. Chem. Eng. Data.

[31] Brunner G., Machado N. (2012). Process design methodology for fractionation of fatty acids from palm fatty acid distillates in countercurrent packed columns with supercritical CO_2_. J. Supercrit. Fluids.

[32] Huang Y., Zhang T., Zhao Y., Zhou H., Tang G., Fillet M., Crommen J., Jiang Z. (2017). Simultaneous analysis of nucleobases, nucleosides and ginsenosides in ginseng extracts using supercritical fluid chromatography coupled with single quadrupole mass spectrometry. J. Pharm. Biomed. Anal..

[33] Samimi R., Xu W.Z., AlSharari Q., Charpentier P.A. (2014). Supercritical fluid chromatography of North American ginseng extract. J. Supercrit. Fluids.

[34] Xie Y.-Y., Luo D., Cheng Y.-J., Ma J.-F., Wang Y.-M., Liang Q., Luo G.-A. (2012). Steaming-induced chemical transformations and holistic quality assessment of red ginseng derived from *Panax ginseng* by means of HPLC-ESI-MS/MSn-based multicomponent quantification fingerprint. J. Agric. Food Chem..

[35] Yang H., Lee N.Y., Kang K.B., Kim J.Y., Kim S.O., Yoo Y.H., Sung S.H. (2015). Identification of ginsenoside markers from dry purified extract of *Panax ginseng* by a dereplication approach and UPLC–QTOF/MS analysis. J. Pharm. Biomed. Anal..

[36] Lee J., Choi B.-R., Kim Y.-C., Choi D.J., Lee Y.-S., Kim G.-S., Baek N.-I., Kim S.-Y., Lee D. (2017). Comprehensive profiling and quantification of ginsenosides in the root, stem, leaf, and berry of *Panax ginseng* by UPLC-QTOF/MS. Molecules.

[37] Chen Y., Zhao Z., Chen H., Yi T., Qin M., Liang Z. (2014). Chemical differentiation and quality evaluation of commercial Asian and American ginsengs based on a UHPLC-QTOF/MS/MS metabolomics approach. Phytochem. Anal..

[38] Wang H.-P., Zhang Y.-B., Yang X., Zhao D., Wang Y.-P. (2015). Rapid characterization of ginsenosides in the roots and rhizomes of *Panax ginseng* by UPLC-DAD-QTOF-MS/MS and simultaneous determination of 19 ginsenosides by HPLC-ESI-MS. J. Ginseng Res..

[39] Li F., Lv C., Li Q., Wang J., Song D., Liu P., Zhang D., Lu J. (2016). Chemical and bioactive comparison of flowers of *Panax ginseng* Meyer, *Panax quinquefolius* L., and *Panax notoginseng* Burk. J. Ginseng Res..

[40] Liu S., Cui M., Liu Z., Song F., Mo W. (2004). Structural analysis of saponins from medicinal herbs using electrospray ionization tandem mass spectrometry. J. Am. Soc. Mass Spectrom..

[41] Zhou Q.-L., Zhu D.-N., Yang X., Xu W., Wang Y.-P. (2018). Development and validation of a UFLC–MS/MS method for simultaneous quantification of sixty-six saponins and their six aglycones: Application to comparative analysis of red ginseng and white ginseng. J. Pharm. Biomed. Anal..

[42] Karpova R.V., Shevchenko V.E., Bocharov E.V., Sheychenko O.P., Bocharova O.A., Kucheryanu V.G., Bykov V.A. (2016). Ginsenosides definition in plant extracts by means of high through liquid chromatography with tandem mass-spectrometry. Russ. J. Biother..

[43] Li S.-L., Lai S.-F., Song J.-Z., Qiao C.-F., Liu X., Zhou Y., Cai H., Cai B., Xu H.-X. (2010). Decocting-induced chemical transformations and global quality of Du–Shen–Tang, the decoction of ginseng evaluated by UPLC–Q-TOF-MS/MS based chemical profiling approach. J. Pharm. Biomed. Anal..

[44] (2008). Russia State Pharmacopoeia XII.

